# Young people’s proposals for a web-based intervention for sexual health promotion: a French qualitative study

**DOI:** 10.1186/s12889-023-16257-8

**Published:** 2023-07-19

**Authors:** Philippe Martin, Corinne Alberti, Serge Gottot, Aurélie Bourmaud, Elise de La Rochebrochard

**Affiliations:** 1 Université Paris Cité, ECEVE, Inserm U1123 UFR de Médecine, 10 Avenue de Verdun, 75010 Paris, France; 2 AP-HP, Hôpital Universitaire Robert Debré, Unité d’épidémiologie clinique, 1426 Inserm, CIC France; 3 GDID Santé, Paris, France; 4 Institut National d’Etudes Démographiques (INED), UR14 – Sexual and Reproductive Health and Rights, Aubervilliers, F-93322 France; 5 Université Paris-Saclay, Univ. Paris-Sud, UVSQ, CESP, INSERM, Villejuif, F-94800 France

**Keywords:** Health promotion, Internet, Sexual health, Young people, Participatory intervention.

## Abstract

**Background:**

Promoting sexual health is key to improving the supportive behaviors and well-being of young people. With the advent of the Internet, web-based features for sexual health promotion may be attractive to a diverse range of young people. This study aims to assess young people’s proposals regarding a web-based intervention for sexual health promotion.

**Methods:**

Nineteen French young people aged 15–24 years participated to the study. In a semi-structured interview, they presented their views on a web-based intervention for sexual promotion. Data were coded with N’Vivo and subjected to qualitative thematic analysis to explore their proposals.

**Results:**

The majority of participants (n = 18) thought that a web-based intervention for sexual health promotion would be attractive. Young people interviewed made 31 concrete proposals for sexual health promotion on the Internet. Participatory and interactive dimensions on the internet appeared essential, with the need for stimulating activities and interaction with peers, but also with competent professionals and moderation. Face to the risks of the internet, they expressed the need of a secure and confidential space, to generate trust and participation in intervention. For participants, sexual health should be addressed in all its dimensions, taking into account the relational, sexual, and gender dimensions, and by incrementing on the internet valid, credible and personalized content.

**Conclusions:**

In sexual health promotion, young people are indispensable stakeholders who can make concrete proposals and can also participate in content creation and research. More broadly, in health promotion, involving target audiences in decisions represents a promising perspective.

**Supplementary Information:**

The online version contains supplementary material available at 10.1186/s12889-023-16257-8.

## Background

Emotional and sexual life are a major concern for young people [1]. To help them to increase their empowerment over their sexual health and well-being, interventions for sexual health promotion have been developed. These interventions promote a positive and safe approach to sexuality by considering cognitive, emotional, social, interactive and physical aspects of sexuality [2]. Based on human rights and gender equality, interventions for sexual health promotion aim to help young people to develop the knowledge, skills, attitudes and values to empower them to thrive and to develop respectful and fulfilling relationships [3]. Comprehensive sexuality education remains essential, addressing all dimensions of sexual health [3]. However, there are still few initiatives or they do not reach young people, and even more so the priority and vulnerable groups (isolated young people, those affected by HIV) [1, 4]. Traditional sexuality education methods were initially based on risk and biological aspects, whereas today’s sexuality educators have adapted their content to take a more global approach to emotional and sexual life, thus meeting young people’s expectations [5].

Young people are born into the digital world, with access to the Internet and cell phones, becoming an integral part of their intellectual and social life [1]. Given young people’s daily digital uses, web-based and digital interventions can be a new way to reach young people for sexual health promotion [6], with facilitated ways to needed information and to empower these young people [7]. To this, the literature shows a diverse panorama of digital platforms used for educational interventions in youth sexual health promotion (e.g., websites, social media, games, apps, text messaging and mailing) [8]. There is a wealth of information on the design and implementation of Internet and digital interventions [9–12], with a wide variety of designs, content and functions. Interventions, in their media and functionalities, are developing, for example, dedicated platforms, the use of SMS or instant messaging, the use of webisodes of media series, the use of online social networks or live chat [13].

Internet and digital interventions have been on the scene for several years now, starting with the simple online transmission of information, making it difficult to find and sort reliable and valid information [5]. Over time, and with the evolution of digital technology, many existing interventions have taken advantage of these tools to promote social interaction and participation in sexuality education, in line with young people’s expectations [11, 12, 14]. Today’s digital features make it easy for young people to interact and participate in sexuality education [9]. This is in line with UNESCO’s recommendations for a comprehensive, participatory approach to comprehensive sexuality education [3]. The Internet and digital technology are then resources that favor portability, anonymity, informality, “personalized” responses or the possibility of interacting with peers who are not in the immediate geographical area [15], especially on these intimate and sensitive topics. In addition, social networking features allow for social interaction and youth engagement in sexual health promotion [16]. The participatory and interactive dimensions, such as play activities or professional and peer support, appear as potential actions to be studied further [9].

Research still needs to better develop data on conception and implementation conditions for these web-based interventions for sexual health promotion [17], as these conditions may influence the effectiveness of these interventions [18]. This implies analyzing which digital interventions are innovative and relevant for the promotion of young people’s sexual health, particularly in light of the rapid obsolescence of digital tools and the constant evolution of uses [9, 19] and existing educational strategies for health promotion [20].

Recent literature reviews, for example, highlight the theoretical and practical underpinnings in the design of digital sexual health promotion interventions. Most interventions are generally built on learning and behavioral theories. The approaches of social learning, individual skills, behavior change and motivation are generally mobilized in the field of sexual health [5, 9, 21].

Also, the participatory approach to intervention design is present in many interventions [9, 21], generally based on user feedback and less on more central participatory design [21]. The participative approach is emphasized both at the level of action [9] but also on how to involve young people in sex education research [10]. The findings on expectations and existing interventions highlight different ways of developing sexual health promotion via the Internet and digital, with the need to integrate an understanding of community expectations.

In this context, several studies have been carried out on young people’s needs and expectations. A qualitative study highlighted some of young people’s expectations, particularly with regard to the digital dimensions of sexual health [22]. Privacy and confidentiality are important dimensions for young people. Beyond ergonomics, the credibility and trustworthiness of interventions are essential [5, 22]. These elements are in line with expert advice on understanding, preserving and maintaining young people’s digital privacy [10]. A review of qualitative studies addresses the issue of young people from sexual minorities. In addition to a comprehensive approach to sexual health, it shows that young people feel there is still a lack of relevant content on sexual health education, including information on same-sex sexual behavior, sexual orientation and gender identity. This highlights the importance of an inclusive approach expected by all stakeholders [9, 10, 23].

For it, taking into account young people’s points of view would be in line with the recommendations of UNESCO, which underline the importance of enabling young people to lead, advise on and influence the content and delivery of digital sexual health promotion [17]. Web-based interventions would be more relevant if based on concrete proposals, upstream of development and linked to pragmatic intervention components. Involving young users in the design of this type of intervention is essential to identifying and understanding their needs, whether in terms of sexual health content or technical medium [19]. Listening to and satisfying young people’s desires in terms of design and content are essential to attract them [6, 24].

This study aims to assess and understand young people’s concrete proposals for a web-based intervention for sexual health promotion.

## Methods

We conducted a qualitative study with young people aged 15 to 24 years living in France to explore their proposals for a web-based intervention for sexual health promotion. This qualitative study is based on a phenomenological approach, since the aim is to directly describe the experiential processes involved in using digital technology to promote sexual health. It places young people’s new and current uses at the heart of the analysis, with the aim of understanding the responses adapted to promote sexual health according to these uses. This paper is reported in accordance with the Consolidated Criteria for Reporting Qualitative Research [25] (see Supplementary Material 1). The study obtained a favorable opinion (n°18–515) from the Comité d’évaluation éthique de l’Inserm (CEEI-IRB0000003888) [26] and was reported to the Inserm Data Protection Officer, with respect to Declaration of Helsinki.

### Inclusion criteria

The inclusion criteria were as follows: (1) young people aged 15 to 24, (2) French-speaking, (3) living in France, (4) general or specific population. People were excluded if they didn’t meet all these criteria (adults outside the age range, non-French speakers, not living in France).

### Recruitment of participants

The recruitment of participants was carried out between 2019 and 2020. Participants were recruited through information notice distributed by health and educational professional partners, social networks sites and researchers’ network. The participants were mainly recruited mainly outside the healthcare structures (at school, in associations). These different relay modes were present in different French regions. We relayed a flyer informing about the study and its objective by forwarding it to a middle school teacher to recruit teenagers, to integration aid organizations to reach out-of-school youth, and through the network of educational and health professionals throughout France to get different young people in different territories. We also published a post on the study’s Instagram account, this social networking site being one of the most used by young people.

The information notice contained explanations on study (purpose, health theme, measures to guarantee confidentiality and security) and invited volunteers to contact the PM researcher.

### Informed consent

A study information note was provided at the time of first contact with the participant and prior to the interview. To allow the participant to be more comfortable, participants can choice to be interviewed by phone or face-to-face. The authors certify that they have obtained the oral consent of each participant.

Each interview began with an introduction. First, the interviewer (first author of this article, PM, male, cisgender, 30 years old) presented himself as a PhD candidate in public health. Then, the interviewer specified that we wanted to develop a “tool on the internet to help young people in their sexual health”. In line with the WHO framework, he indicated that “sexual health” was defined as all dimensions related to emotional, relational, sexual and reproductive life, which may include sexual and romantic relationships, contraception, sexually transmitted infections and means of protection. He emphasized that the purpose of the interview was to have the point of view of the young on this project and to discuss his/her recommendations to be useful and attractive. Finally, he checked if the participant had any question and whether he had read the information note.

In accordance with French regulations, the interviewer requested oral informed consent to participate to the study. Participants who were minors did not need parental authorization, according to the French regulations in force, as they were at least 15 years old. The Comité d’évaluation éthique de l’Inserm (CEEI-IRB0000003888) validated the following point: the participation in our survey falls within the autonomous competence of the minor (sexual and numerical majority, sexual and reproductive rights as anonymity for access to some sexual health services). This position is based on i) the notion of “acts of everyday life” for which case law recognizes the autonomy of the minor. It is also based on ii) the International Convention on the Rights of the Child of November 20, 1989 [27], a United Nations convention to which France is a State Party, which states that the child is free to express himself and therefore to give his opinion. On this basis, Institut national de la santé et de la recherche médicale (Inserm) and Institut national d’études démographiques (Ined) have already carried out, under similar conditions, surveys on sexual health including minors (FECOND study, Inserm - Ined, 2010 [28]). For this study, the CEEI-IRB has approved oral informed consent for any participant 15 or older (without parental consent for minors), as mentioned in the research protocol and having received a favorable opinion. Nevertheless, the interviewer suggested that they informed their parents of their participation in the study.

The interviewer asked if the young gave permission to record his/her interview on audio tape or if he/she preferred to be interviewed without being recorded. Finally, the interviewer specified that the young should feel free during the interview to dismiss any question or topic that he/she would not feel comfortable with.

### Semi-structured interviews

The interviewer PM conducted interviews. He had degrees in public health (PhD) and sexual health (university diploma), with training in the techniques of conducting individual and group interviews.

A semi-structured topic guide shaped interviews. The interview guide was developed in collaboration with all the authors specializing in qualitative research, sexual health and youth health. The interview guide was developed, adapted and validated by all the authors. It is structured around elements of the participants’ characteristics, contextual elements around sexual health and concrete proposals for action. As part of the usual qualitative research approach, the interview guide was tested and then adapted after the first interviews with the young people. The questions were open-ended and neutral, in order to capture the young people’s views as fully as possible. For each question, the interviewer was able to prompt the participants for further information. The final version of the interview guide is available in Supplementary Material 2.

The first part of the interview aimed to initiate discussion with introductory questions on sexual health concerns, use of the Internet (in general and for sexual health concerns) and resource people for sexual health.

Then, the interviewer asked: “*If an action were to be carried out on the Internet, for young people and for sexual health: how would you imagine it?*“. This initial large question aimed to collect concrete proposals of the young on the web-based intervention for sexual health promotion.

Depending on the interviewee’s response, the researcher asked more precise questions in order to address the different issues: sexual health topics to address, online formats, activities, functionalities, and general operation that could be attractive for these topics.

### Analysis

Digitized interview transcripts and notes taken during the interviews were transcribed with anonymization of all identifying information (PM). Then, a thematic analysis was carried out with NVivo10 software, by two authors (PM, ELR) who followed the recommended steps for the development of the themes in terms of qualitative analysis, here focus on proposals identification and analysis (Initialization, Construction, Rectification, and Finalization) [29]. The initialization phase corresponded to an initial description of the participants’ various raw propositions. The PM researcher took notes on emerging themes and categories that might emerge from the concrete proposals. The two researchers, PM and ELR, carried out the construction phase, classifying and describing the participants’ various proposals using a classification system. For the analysis, each proposition was associated with contextual elements linked to affective and sexual life as put forward by the participants. This made it possible to better contextualize the analyzed propositions. The rectification phase corresponded to the distancing of the themes, notably by reorganizing and reformulating the themes around areas linked to digital health promotion actions. This phase enabled us to highlight those themes that provided genuinely new scientific knowledge in relation to existing theory. Finally, the finalization phase allowed us to set the themes according to the link or distance between them (fluidity for understanding the proposals).

The transcripts were coded (PM, ELR) using an inductive proposal identification process. Based on analysis consensus among authors, proposals were then grouped into main themes to develop a web-based intervention for sexual health promotion.

## Results

Nineteen young people were interviewed in 2019–2020. They were mainly informed of the study by health and educational professional partners (n = 11), most of the other were informed through researchers’ network (n = 3), word of mouth from the firsts participants themselves to their friends or peers (n = 4), and only one thanks to social networks site Instagram. Interviews were mainly carried out by phone (n = 14), with few interviews carried out face-to-face in a coffee shop (n = 4) or at the researcher’s workplace (n = 1).

Detailed description of participants is presented in Table 1. The majority of participants were young adults between 18 and 24 years old (14/19), women (12/19), students (12/19), living in the Paris area (11/19). Nine participants declared themselves to be heterosexual, four declared themselves to belong to an LGBT + orientation, six did not mention their sexual orientation.

**Table 1:** Participants characteristics (n = 19)

ID	Age	Gender*	Sexual orientation declared*	Professional status	Region of residence
A1	20	Male	Heterosexual	University student	West of France
A2	21	Female	Not specified	University student	Paris region
A3	23	Female	Heterosexual	No activity	Paris region
A4	23	Male	Heterosexual	In employment	Paris region
A5	24	Male	Homosexual	In employment	Paris region
A6	16	Female	Not specified	High school student	West of France
A7	23	Male	Homosexual	University student	Paris region
A8	24	Male	Heterosexual	In employment	Paris region
A9	24	Female	Heterosexual	In employment	Paris region
A10	19	Female	Bisexual	University student	West of France
A11	19	Female	Heterosexual	University student	West of France
A12	24	Female	Heterosexual	In employment	West of France
A13	23	Female	Not specified	In employment	Paris region
A14	20	Female	Heterosexual	University student	West of France
A15	16	Female to Male	Pansexual	High school student	Est of France
A16	24	Female	Heterosexual	University student	West of France
A17	15	Female	Not specified	College student	Paris region
A18	15	Male	Not specified	College student	Paris region
A19	15	Female	Not specified	College student	Paris region
**Living place**					
Family Home		10		53%	
Personal apartment		9		47%	
**Interview duration (minutes)**					
≤ 29		3		16%	
30–59		7		37%	
60–90		9		47%	

* We asked for the gender at the beginning of the interview. We noted information about sexual orientation only if the participant mentioned it during the interview (not requested)

All participants use the Internet on a daily basis. They had a smartphone allowing them to access web-based content anywhere. Most of them used Internet primarily to search divertissement and social interaction with friends known offline. They also used the Internet for their student or professional work, by searching online information on websites or on specialized platforms. A majority of participants (n = 18) reported using SNS for divertissement, mainly Snapchat, Instagram and YouTube. One participant reported using Discord to chat on video games (A8).

The majority of participants (n = 18) found attractive the project of a web-based intervention of sexual health promotion. One participant did not find it attractive, because the theme is too intimate for him to be engaged in a specific and formal intervention. At the end of the interview, the majority of participants (n = 17) indicated that they could provide input or participate in the development of a concrete tool.

The thematic analysis highlighted five main themes to develop a web-based intervention for sexual health promotion: (A) proposals to promote sexual health in web-based intervention; (B) proposals for a trustworthy web-based intervention; (C) proposals for an attractive and accessible web-based intervention; (D) proposals for personalized, participatory and interactive web-based intervention; and (E) proposals for a safe and confident web-based intervention. These main themes integrate a total of 31 concrete youth’s proposals. These themes and proposals are presented in Table 2.

**Table 2:** Young people’s proposals for a web-based participatory intervention for sexual health

Main theme	Proposal number	Proposal description
A - Promote sexual health in web-based intervention	1	to address biological and medical questions on sexual health, including body and anatomic changing during puberty, contraception, HIV but also other sexually transmitted diseases
	2	to address sexuality in general, notably addressing relationships, pleasure, not just health
	3	to address concerns structured by personal experience much more than by age
	4	to promote sexual health outside the “norm” of heterosexuality (including sexual orientation and transidentity) of young people in good health
B - Trustworthy web-based intervention	5	to provide centralized and credible information
	6	to involve specialized professionals to promote sexual health in the web-based intervention to be able to provide trustworthy information
C - Attractive and accessible web-based intervention	7	to provide playfulness information on sexual health
	8	to be fun, non-infantile, unformatted and non-institutionalized
	9	to permit to navigate easily in the web-based intervention thanks to a good general organization with information section according to type of content (information, interaction, and help numbers)
	10	to propose different explicit categories on sexual health: types of sexual health issues, types of sexual life situations possibilities, types of practices and risks associated
	11	to increment a tweeting system (hashtag) to find more easily resources
	12	to develop a mobile application as it is more accessible on smartphone
	13	to develop a web-based intervention linked (but separated) to preferred uses
	14	to use major media and SNS to advertise on the web-based intervention
	15	to choose a name of the web-based intervention that should be easily identifiable and pronounceable
D - Personalized, participatory and interactive web-based intervention	16	to give an access to ask question to have personalized answers
	17	to publish anonymously recurrent questions and answers, to show to young people that they are not alone and preserve discretion
	18	to give geo-localized resources in the web-based intervention to help access to health services close to where young people live (abortion, STIs)
	19	to offer a vote system on the topics to be discussed or addressed in intervention, because this system will answer to expressed youth’s needs
	20	to implement a serious game, with challenges in life situations, in order to engage participation and projection of participant to anticipate sexual health situations
	21	to give access to quizzes, with complementary information, in order to get information and better understanding, allowing young people to test their knowledge
	22	to generate discussions about daily life situations, because it is important to be grounded in the realities of youth
	23	to reuse elements of youth culture (media-series) to bring about discussion important sexual health topics
	24	to include influencers and the opportunity to interact with them
	25	to give opportunity to interact directly with sexual health specialists and professionals to have highly valid and personalized answers
	26	to permit in intervention peer-to-peer experiences sharing through discussion forums or chats to feel less alone in life situations including sexual health
E - Safe and confident web-based intervention	27	to propose a proper supervision of all exchanges with an efficient system of moderation
	28	to provide individual and personal responses in private through e-mails or telephone responses, in order to discuss topics confidentially with knowledgeable people
	29	to separate web-based intervention from SNSs, in order to have a watertight seal between the identifying networks and their intimate spheres on the Internet
	30	to permit online anonymity for sexuality issues, especially for intimate matters related to sexuality
	31	to permit for participants the use of pseudonyms

### Young people’s proposals to promote sexual health in web-based intervention

Interestingly, only three participants proposed to address biological and medical questions on sexual health. It included interest for body and anatomic changing during puberty (A8, A19), contraception (A9), HIV but also other sexually transmitted diseases (A1, A19) (**proposal 1**). Most participants put forward non-biological sexual health topics.

One participant (A3) proposed to address sexuality in general, notably addressing relationships, pleasure, not just health (**proposal 2**). Three female participants (A3, A10, A14) emphasized the notion of girl’s sexual pleasure and the need to address it in the web-based intervention. One of them (A10) pointed out differences between girls and boys concerning pleasure, with the need to deconstruct societal norms and representations:

*I know that in pornographic films there’s an image of having to please the man….and I never stopped to ask myself about my own pleasure […] Even female masturbation was something not talked about, although for men it’s more or less accepted as natural. (A10, female, bisexual, 19 years old)*

Three participants (A3, A8, A12) raised the norm and social pressure around sexual health issues. One participants (A8) pointed out the need to have support to deal with the strong pressure to have a “normal” sexuality and “normal concerns”:

*I would really have liked to have someone of my own age […] who could say to me “don’t worry, the questions you have are quite normal, everyone has them too (A8, male heterosexual, 24 years old)*

One participant (A12) noted that SNS reinforces this pressure to be “normal” and even to have an “ideal” life because people present themselves with socially desirable pictures:

*On social networks there is a lot of comparison. You have to know the difference between what are people’s normal daily lives [...] and what they want to be seen (A12, female, heterosexual, 24 years old)*

Age at first sexual relationship is one aspect under scrutiny to be “normal”. One participant (A3) emphasized the need to not consider that someone “should” already had sex because of his/her age and to really anticipate in the web-based intervention that concerns may be structured by personal experience much more than by age (**proposal 3**):

*The transition from teenager to young adult doesn’t always happen at the same time for everyone. And it’s very much in stages, you can experience some things when you are very young or you can have a very late development of sexuality, and you can have questions which might seem to be adolescent when you are 23, or not know about sex because you are still a virgin… Everyone has their own identity! (A3, female heterosexual, 23 years old)*

Participants also pointed out that “normality” also supposed that you are “performant” and already know everything and that should also be fight back in the web-based intervention:

*[…] you felt a sort of obligation to experience sex and to know exactly what to do before you ever did it. Even if it was the first time, you were supposed to know it all already, to be able to do everything, know exactly how to do it etc.[…] there was no more communication with the other person (A3, female heterosexual, 23 years old)*

Participants described as very important to promote sexual health outside the “norm” of heterosexuality of young people in good health (**proposal 4**): “*Everyone needs to be involved*” (A12). Sexual orientation (A15 talked about asexuality) and transidentidy were under consideration even among young that were not personally concerned:

*Then the question of sexual orientation is important. For people who have questions but don’t dare to talk about it. I think there are lots of young people who go on the internet. When I see young Trans people… I think there are gender issues…but also about how to transition, if you have the right to do it, what are our rights etc. (A12, female heterosexual, 24 years old)*

Few participants underlined the difficulty to find information for young people that are not heterosexual, especially if they do not live in a big city:

*I think that could be good, it could allow you to feel less alone in the questions that worry you – for me, typically, about my bisexuality. If there had been an app where I could be anonymous, maybe I would have found out there were a lot of girls of 17 who were asking the same questions as me…..I wouldn’t have had to wait till I went to Paris to tell myself it was normal (A10, female bisexual, 19 years old)*

### Young people’s proposals to build a trustworthy web-based intervention

Most participants were open to a web-based intervention for sexual health promotion as they have already used the Internet at least once for sexual health concerns (sex of the other, sexual relationship, contraception, unspeakable subjects). Nevertheless, they indicated strong concerns on credibility and validity of internet information. They indicated that they ensure quality of information by crosschecking pieces of information found on different Internet places (A1, A13). A participant (A8) explained that he compared, contrasted and supplemented the different information he found on the Internet with his friends offline, particularly in adolescence on issues related to sexuality. Considering this context, participant A13 indicated that the web-based intervention for sexual health could appeared as a good option to provide centralized and credible information provided that it will really demonstrate its credibility (**proposal 5**):

*Yes, because I think that on the internet you can find anything and everything [….] I am thinking of an app which is reliable and would reassure everyone [….] that the source is reliable. (A13, female, not specified)*

Participants indicated that they trust “experts” for sexual health issues, they cited health professionals (A1) and science teachers (A10, A19, A20). Thus, participants suggested to implicate in the content of web-based intervention specialized professionals to promote sexual health to be able to provide trustworthy information (A1, A8, A12, and A13) (**proposal 6**).

*To have a health expert I think would be really useful, because you can search all you like on the internet or ask people who are no older than you are yourself, you will never get the kind of answer you can get from someone who is professionally qualified (A1, male, heterosexual, 20 years old)*

### Young people’s proposals to have an attractive and accessible web-based intervention

Participants expressed the need to provide playfulness information on sexual health (**proposal 7**). A participant (A6) indicated that she find written and lengthy content boring:

*Easy to use, [….] you could have a video, click on it easily, instead of having to read, and it’s easier to understand* » *(A6)*

On a practical point of view, participants advised to be fun (A3), non-infantile (A15) unformatted and non-institutionalized (A3) (**proposal 8**):

*It has to be fun, relevant and professional, attractive, easy to access and there shouldn’t be too much formatting. You shouldn’t feel you are going on a WHO website and have to go and look for written information in small print by someone you have never heard of and which is impossible to read (A3, female, heterosexual, 23 years old)*

Two participants (A3 and A6) discussed the importance to permit to navigate easily in the web-based intervention thanks to a good general organization with information section according to type of content (information, interaction, and help numbers) (**proposal 9**). To access easily to content, one participant (A3) insisted on the need to propose different explicit categories on sexual health: types of sexual health issues, types of sexual life situations possibilities, types of practices and risks associated (**proposal 10**). Another (A12) proposed to increment a tweeting system (hashtag) to find more easily resources (**proposal 11**).

Participants (A3, A13) indicated that a website would not be fit to their digital behavior. They proposed to develop a mobile application as it is more accessible on smartphone (**proposal 12**):

*I find it should be an app you can go on more easily than on an internet site where you have to open the page and type in the name of the site (A3, female, heterosexual, 23 years old)*

These participants also underlined the need to develop a web-based intervention linked (but separated) to preferred uses (**proposal 13**): other external digital or web systems as social network sites, applications or others Internet supports.

Two participants also had proposals to insure communication on the web-based intervention. To reach a large number of young people, a participant (A1) suggested to use major media and SNS to advertise on the web-based intervention (**proposal 14**). Another participant (A13) emphasized the importance of the name of the web-based intervention that should be easily identifiable and pronounceable (**proposal 15**). She also proposed to name subtly the application to be confidential, because curious eyes may make the intervention’s users uncomfortable if it is too sexual connoted.

### Young people proposals to have a personalized, participatory and interactive web-based intervention

One participant (A3) proposed to give an access to ask question to have personalized answers (**proposal 16**). Another (A8) also propose to publish anonymously recurrent questions and answers, to show to young people that they are not alone and preserve discretion (**proposal 17**). One participant (A12) suggested to give geo-localized resources in the web-based intervention to help access to health services close to where young people live (abortion, STIs) (**proposal 18**).

Participants (A3, A8, A12, A13) recommended to allow participatory functionalities. They proposed to offer a vote system on the topics to be discussed or addressed in intervention, because this system will answer to expressed youth’s needs (**proposal 19**). A participant (A3) imagined the possibility to implement a serious game, with challenges in life situations, in order to engage participation and projection of participant to anticipate sexual health situations (**proposal 20**). Another participant (A8) propose to give access to quizzes, with complementary information, in order to get information and better understanding, allowing young people to test their knowledge (**proposal 21**):

*A quiz, I think that wouldn’t be much use if it just came to an end. I think you would have to go further: if you give a wrong answer it’s because you didn’t know, but the right answer is this (A8, male, heterosexual).*

Participants evoked social interactions in intervention. To promote interaction, a participant (A3) highlighted the need to generate discussions about daily life situations, because it is important to be grounded in the realities of youth (**proposal 22**). Another participant (A12) proposed to reuse elements of youth culture (media-series), to bring about discussion important sexual health topics (**proposal 23**).

Two participants (A6, A10) proposed to include influencers and the opportunity to interact with them (**proposal 24**). They considered influencers funny and entertaining, and they can give lifestyle advice with humor. Five participants (A6, A10, A12, A18, and A20) indicated follow influencers on Social Network Sites (SNS), presents on YouTube, Instagram and Snapchat, sometimes with excerpts from real-TV shows. Three participants (A3, A10, and A15) also declared that they follow SNS accounts committed to sexual health (sexual and gender minority rights, feminism).

For one participant (A10), an influencer can be also an advocate: “*He makes me laugh, I saw he had made a campaign on harassment. He went to see the minister to talk about harassment and so on. So, he’s funny but he also has ideas that appeal to me a lot*” (A10, female, bisexual, 19 years old). One participant (A6) explained that she can learn many things from an influencer, whom she admires and trusts more than other standard websites offering information: “*Google isn’t the same because you don’t really know who is giving advice, but when you go on Instagram and find a Youtuber, you know, you can trust her more”* (A6, female, 16 years old).

Concerning interaction, another participant (A12) proposed to give opportunity to interact directly with sexual health specialists and professionals, because they are health experts and will be able to give highly valid and personalized answers to young people (**proposal 25**) (link to propositions to trust in intervention). Participants (A3, A8, and A10) proposed to permit in intervention peer-to-peer experiences sharing through discussion forums or chats, because it helps people feel less alone in life situations including sexual health (**proposal 26**). More globally, peers interactions then appeared attractive for intervention, with conditions: “*Yes, if there are young people involved in the project that gives it an added value. But then, it all depends how!” (A3, female, heterosexual, 23 years old).*

In the web-based intervention, the interest for interactions between participants should probably be considered more specifically for minorities. Four participants from minorities (A5, A7, A10, and A15) - who identified themselves as gay, lesbian, trans or pansexual - indicated that they use Internet to get in touch with peers of gender or sexual identities to have interaction with people “*just like them*” (as they had none in their friends group in physical life). A participant specified that its goal was to only exchange and not especially to engage into sexual relationship:

*I wanted to talk about it with people who were similar to me, like with other gays, and for me it was mostly about contact apps […] it wasn’t sexual, it was just to be able to communicate with people my own age (A7, male homosexual, 23 years old)*

### Young people’s proposals to have a safe and confident web-based intervention

Participants (A5, A10, A12) warmed that sexuality remained taboo and very difficult to talk about, even on the internet. Thus, they underlined the need to really work on building a web-based space facilitating openness:

*I think it’s a brilliant idea. But then there’s the question of how to lead into the subject because the issue of sexuality is rather taboo [….] you need a good way to introduce it… (A12, female heterosexual, 24 years old)*

All the participants highlighted the Internet risks, in general and for the web-based intervention: cyber stalking, exposure of personal and nude images, disclosure of intimate feelings, predators, addiction, and comparisons to others.

*The internet is great but I think it may encourage harassment. You are hidden by a screen and there’s a group effect on behaviour. [….] The internet makes it easier to pass judgement on other people, so it can quickly get out of hand in colleges and schools and so on. (A10, female, bisexual, 19 years old)*

Because of these risks, most of the participants indicated that they do not contact or interact with people they do not know. Participants were thus both attracted but worried on the idea to exchange with other young peoples in the web-based intervention. They emphasized the importance to propose a proper supervision of all exchanges with an efficient system of moderation (**proposal 27**). For a participant (A3), position of moderators has to be an appropriate balance between controlling excesses and ensuring freedom of speech:

*The positioning [of moderator] is going to be really important. There shouldn’t be too many presumptions, you shouldn’t find it too strict but also you shouldn’t have the feeling that it’s too loose [….] there should be a dialogue that’s free, open and really liberated and which is useful… (A3, female, heterosexual, 23 years old)*

When discretion is required on a topic, one participant (A13) proposed to provide individual and personal responses in private through e-mails or telephone responses, in order to discuss topics confidentially with knowledgeable people (**proposal 28**).

Moreover, a participant (A10) explained she compartmentalize social interactions online, with the example of using Facebook to communicate with her family, while Snapchat is preferred for chatting with friends. Another participant (A3) emphasized the need to compartmentalize sexual concerns navigations on the Internet from common SNSs uses, for which all are recognizable. She proposed to separate web-based intervention from SNSs, in order to have a watertight seal between the identifying networks and their intimate spheres on the Internet (**proposal 29**): “*a system of profiles which would not be related to social media like Facebook or Instagram, so as to remain in a separate compartment”* (A3).

In this sense, participants (A3, A10, A13) expressed the need to permit online anonymity for sexuality issues, especially for intimate matters related to sexuality (**proposal 30**), because it is necessary to talk about these intimate subjects in a safe and confidential way, without being recognized.

*For me, anonymity (in sexual health matters) is super important. There needs to be no leakage between that and what is visible for all my friends on Facebook, through work etc. (A3, female, heterosexual, 23 years old).*

To preserve anonymity in an intervention, one participant (A14) suggested to permit for participants the use of pseudonyms (**proposal 31**).

## Discussion

Our study highlights young people’s concrete proposals for a web-based intervention for sexual health promotion. The results of the study meet the main objective of investigating young people’s expectations and concrete proposals for the promotion of young people’s sexual health, taking into account current sexual health contexts and promising digital functionalities to be implemented. This leads to several points of discussion that we would like to raise.

Firstly, participatory and interactive dimensions on the internet appeared essential for participants, with fun activities and peer interactions, but also with competent professionals to increment valid, credible and personalized content. Secondly, face to sensitive subjects, participants need a moderate, secure, and confidential space, to trust and participate. Thirdly, sexual health should be addressed holistically and correctly, taking into account the emotional, sexual, and gender dimensions.

### Ensure participatory, interactive and ergonomic features to enroll young people

Study participants made more than ten proposals in favor of participatory and interactive components, in order to be engaged in a web-based sexual health promotion intervention. These proposals are in line with the World Health Organization recommendation to implement participatory sexual education [2]. To this end, participants proposed concrete and engaging activities such as vote system, quizzes or games. This is in line with other studies that have identified the levers to engage young people, such as attractive functionalities: interactive quizzes, games [30, 31], decisional activities [6]. These decision activities highlighting the need of intervention components that could be personalized, user-centered beyond participation only. Our participants proposed personalized answers to each individual questions and an access to geolocated resources close to the participants’ living areas. Beyond functionalities, they also propose to address content on daily life situations.

This personalization can occur around the sharing of common individual experiences. For study participants, interactions between peers and with professionals appeared a way to enroll and involve them. This result is in line with a previous study in UK, exploring 67 young people’s attempts at sexual health promotion, showing that young people want social interaction online and to see peers points of views [6]. Peer interaction, by exchanging information, knowledge, experiences and sharing common values, can be a way to education [32–34]. Discussions, debates and reflections can enable empowerment, beyond passive information. Fostering interaction requires a regular and constant presence and responsiveness. Based on social media analyses, a previous study has highlighted key aspects for engagement in interactive health promotion intervention: regular publication of messages, reactivity and positive reaction to participants’ messages, in addition to relevant content [35]. Actors in charge of animation can include individual responses, approve by “likes”, encouraging interaction through questions, or involving celebrities (influencers) [20, 35, 36]. High reactivity and an adequate response are then the keys for success. However, interactivity is not always a lever. A paradox expressed in our study is the need for interaction and experience sharing, and in the same time, the general refusal to talk or express oneself freely with strangers on the Internet. This can be set against the categories of online profiles shown in the literature. Indeed, the research literature has shown that Internet users are more lurkers than generators of contents or discussions [37]. Other solitary participatory activities could then engage these young people who don’t want to expose themselves or interact on the Internet.

More globally, to implement participatory and interactive feature, the participants interviewed in our study emphasized the need for tools that are fun, functional, easy-to-use and adapted to their current Internet uses and practices. This is in line with previous study, highlighting the interest of ergonomic formats (images, videos) [9, 38]. A study have highlighted young people’s expectations for online or digital sexual health promotion interventions, notably the need to address all themes, through social interaction and illustrative media such as images and videos [6]. User-friendly aspects adapted to youth culture are also levers of engagement [9], notably to avoid boredom. Indeed, young people seeking health information on the Internet may be strongly influenced by the look and feel of a web resource [24]. Beyond the ergonomics and attractive dimensions, young people value easily understandable content, a clear layout of the information and the credibility of the content publisher [39]. As we highlighted, a study shown that an intervention was considered trustworthy and credible by youth because there is evidence-based content and nonjudgmental tone [40]. The assurance of reliable and credible content is therefore essential, with the involvement of knowledgeable professionals. In this sense, the participants also emphasized the need for confidence in this kind of intervention.

### Ensure security and confidentiality to speak about sensitive subjects

Participants explained that real engagement in intervention depends on credibility content and how health promotion actors secure interactions on the Internet. In order to engage them, participants emphasized the need for a safe, anonymous and moderate online space, especially for intimate issues. Study participants indicated that they compartmentalize their intimate lives related to sexual health on the Internet. They want to separate elements of their intimate lives from identifying social networks. It is in line with a qualitative US study with eight young women explaining that it remains difficult for them to show on social networks central elements of oneself such as sexual identity [41]. Although Internet is a good medium to anonymously address very sensitive sexual health topics, engagement in intervention is then dependent on the confidence that young people have in a secure web-based space.

Sexual health topics have already been considered as particularly intimate and sensitive in previous studies conducted among German and US adolescents [39, 42]. A recent study highlights that emotions, particularly shame and embarrassment, shape young people’s access to and use of sexual health information and may lead them to seek out resources online [43]. To address this embarrassment, study participants made a proposal: provide a portal to ask questions anonymously to address the diversity of topics. This is in link to a qualitative US study analysing common questions of young people in sexual health [44]. In addition, another Australian study highlights that sharing experiences among peers can be attractive, but with the risk of being revealed: young people need to remain fully anonymous to preserve privacy [40]. Indeed, a qualitative study on 49 Scottish participants (16–19 years old) highlighted that young people may be opposed to engaging with sexual health promotion online content, because they are visible and could be judged by others or discovered in “real” life [38]. Another a study in all Africa highlights that youth were more likely to engage superficially with peer-generated sexual health messages using feedback rather than comments or sharing messages with members of their network [45]. An anonymous intervention should then allow for confidentiality, especially if interactions are possible in intervention. To this end, our participants also added the value of using pseudonyms to preserve anonymity.

Moreover, while interesting, study participants pointed out others potential pitfalls of online interactions. A second level of security’s intervention is then the moderation. Face to online interaction risks (false information, cyberbullying), a previous US study has shown that individuals are more likely to participate in online interactions if there are signs of moderation [46]. Although it is costly, moderators must be trained both in the technicalities of moderation, but also be sufficiently knowledgeable in sexual health to have an appropriate response. Building confidence to reach and engage young people requires time and must be able to deal with rapidly changing uses, diverse questionings and priorities. Some young people are in complex trajectories, especially in terms of sexual health. They may be at risk of being rejected by others or by themselves [20]. This confidence in the intervention could intervene beyond online tool, with the need to correctly apprehend the diversity of youth and all of the topics related to the sexual health theme.

### Ensure good values to address all sexual health subjects and concerns

Participants expressed the importance of sexual health in young people’s life. They recalled the diversity of their generation’s trajectories and concerns regarding sexual health. For a web-based intervention, participants highlighted the importance of dealing with these topics related to emotional and sexual life, in addition to diseases. Like our results, another scoping review explained that young people are in favor of a sexual health education that includes pleasure and relationships and is not limited to biology, safe sex and pregnancy [19]. In line with our results, a Dutch study showed that young people still need more sexuality education, beyond biological aspects: sexual consent and coercion, diversity, pleasure, relationships, dating, communication and sex in the media [47]. Sexual health and well-being depend on access to comprehensive, quality information, knowledge, services and supportive environments that promote sexual health [48].

However, another Chilean study shows that few adolescents have received this kind information (emotional-relational aspects) [49], although it was recommended by young people themselves (relationships, breakups, parenting, pleasure) [50]. As stated in the international policy recommendations [2, 3], sexual health promotion is important in a comprehensive, tailored, and holistic approach (sexuality, rights, values, risks). Moreover, during our study, participants highlighted the risks of the Internet in relation to sexual health, particularly with the example of nudes and the online disclosure of bodies and feelings. This brings up new sexual health issues related to the Internet should be taken into account (text pornography, cyber-bullying and nude images) [3, 17].

In addition, our participants proposed to have a holistic but also inclusive approach in intervention, particularly in terms of gender and sexual identities. WHO and authors recommended involvement of minority groups (LGBTQI+, handicap, diseases), with an inclusive approach and the acknowledgement of different identities and trajectories [51, 52]. However, studies shown that LGBTQ youth think that sex education is exclusive [53], with gender and sexual minorities unrepresented, unsupported, stigmatized, and intimidated [54]. Personalized, inclusive, non-heteronormative information is still lacking [55]. To overcome this, the Internet is then a way to reach out these minority youth. One US study on a web-based intervention indicates three strongest motivations to engage LGBTQI + youth [56]: talking to other, finding a safe space to talk about their identity, connecting with like-minded youth. Participants from another study expressed that an inclusive model would help sexual minority youth themselves feel more informed and in control of their own gender and sexuality [57].

Youth diversity can be complex to consider for health promotion actors. Sexual health issues for young people are in motion and require constant adaptation. Some issues are dependent on several individual or environmental factors and can be linked to other more global issues (societal commitment, mental health, ecology). It reminds us of the importance to leave behind one’s own representations or judgments, in order to apprehend the range of diversity in the young public, as suggested by professionals interviewed in a French qualitative study and discussed by Carrotte et al. in an Australian qualitative study among young adults [20, 58]. To be holistic and inclusive, targeting public participation is also essential to understand all the issues, especially to develop research on health promotion web-based interventions. While it seems evident that young people need to be involved in strategies, the way to do so is not so clear. Young people need to take ownership of interventions beyond simple consultation. They can be indispensable actors in properly reaffirming their sexual rights. More generally, institutional strategies must take into account the current issues among young people, both in terms of their diversity, their issues on sexual health and their link to internet uses, especially if actors want to intervene in the digital and internet spheres.

### Strengths and Limitations

One strength of our work is that it is a study that collects concrete proposals that can be used to develop health promotion web-based interventions (31 concrete proposals referenced in Table 2). While some of the studies focus on young people’s expectations, none of them includes concrete propositions for intervention components and their means of implementation, particularly for promoting young people’s sexual health on the Internet. These proposals could be taken up in whole or in part by the various stakeholders for a health promotion intervention centered on the real uses of young people for their own health.

Another strength of our study is that it allows us to understand the logic and the reflexivity of young people when interacting with internet tools, particularly in a context of rapid evolution of internet and communication technologies. They considered the theme of health beyond their own trajectory. They offered reflections on the internet as a tool with its risks and advantages. They considered what could work for the effective promotion of sexual health on the internet (levers).

Several limitations of the study should be taken into account, in particular concerning the results and their generalizability. A limitation of this study is the lack of participants with special needs and less favorable living conditions. We were not able to recruit young people in precarious situations or with disabilities. Our sample is also more female, which raises questions about differences in the recruitment of young boys or other genders. Nevertheless, we were able to reach different participants who were open to other life situations, thus providing points of analysis of the diversity of the young population. This leads to a reflection on the places of recruitment, for qualitative study and for a web-based intervention, in order to be able to reach the multiple profiles of young people. The solicitation of specialized organizations (associations, social structures) could improve the chances of this, as well as recruitment through social networks [59, 60], to include hard-to-reach populations in a larger sample.

Qualitative interviews were conducted by a male interviewer in his thirties, by telephone, in a public place or in the research laboratory. These interview parameters could have affected the participants’ responses, notably through a social desirability bias. Nevertheless, the researcher probed and rephrased the participants’ answers to ensure that they were correct. Also, the interview setting was that chosen by the participant, to ensure maximum confidentiality and ease in answering the study questions.

The data was collected between 2019 and 2020. The rapid evolution of digital tools is to be considered in the current consideration of concrete proposals for action. However, the concrete proposals address digital-related elements that go beyond the simple rapid obsolescence of these digital tools.

Some optimization points for web-based interventions might not have been addressed by our panel. For example, accessibility was rarely brought up by the participants. Although the young people proposed a wide-ranging communication, none of the proposals addressed how to reach young people with disabilities or who are far from health education and promotion initiatives. This point should be explored further in future studies.

Finally, the participants interviewed had no experience in developing online sexual health promotion activities. However, some of them were very active and proactive outside of formal and institutional interventions. One possibility would have been to recruit young people who have experience of producing online sexual health promotion content, such as influencers on social networks sites. However, this could have had the disadvantage of locking them into their concrete experience, without the possibility of opening up new potentialities for intervention.

## Conclusions

In health promotion, involving young people in decisions through participatory research represents a promising perspective, because they can be considered as credible partners in the intervention research process [61, 62], like any other stakeholder. They can offer indispensable elements that the researchers would not have thought of, especially for the development of web-based interventions for health promotion. Therefore, participatory research carried out with young people makes it possible to orient and structure interventions that meet expectations and needs. In the past, the field of AIDS has shown the advantages of including the target public in the decisions, and has inspired many initiatives in health democracy [63]. Its principle is to place all health actors, including patients or consumers, at the heart of the development of health interventions or policies [64]. For sexual health, such a decision-making process seems a promising avenue, and young people should be considered as indispensable stakeholders in developing relevant sexual health promotion interventions.

## Electronic supplementary material

Below is the link to the electronic supplementary material.

Supplementary material 1: Consolidated criteria for reporting qualitative studies (COREQ): 32-item checklist

Supplementary material 2: Interview guide

## Data Availability

The datasets used and/or analyzed during the current study are available from the corresponding author on reasonable request.

## List of abbreviations

AIDS: Acquired Immuno Deficiency Syndrome
CBPR: Community-based participatory research
CEEI-IRB: Comité d’évaluation éthique de l’Inserm
COREQ: Consolidated criteria for reporting qualitative research Consolidated criteria for reporting qualitative research
HIV: Human Immunodeficiency Virus
Inserm: Institut national de la santé et de la recherche médicale
Ined: Institut national d’études démographiques
LGBTQI+: Lesbian, Gay, Bisexual, Trans, Queer, Intersex and others gender and sexual identities
SNS: Social Networks Sites
UNESCO: United Nations Educational, Scientific and Cultural Organization
US: United States
WHO: World Health Organization
