# Supplementary material for: Young people’s proposals for a web-based intervention for sexual health promotion: a French qualitative study
Source: BMC Public Health. 2023 Jul 19;23:1389. doi: 10.1186/s12889-023-16257-8 (PMC10357632; doi:10.1186/s12889-023-16257-8)
Supplement: Supplementary file 1 — Supplementary material 1: Consolidated criteria for reporting qualitative studies (COREQ): 32-item checklist [file 12889_2023_16257_MOESM1_ESM.docx]

Multimedia Appendix 1: Consolidated criteria for reporting qualitative studies (COREQ): 32-item checklist

| **Domain 1 : Research team and reflexivity** | **Personal characteristics** | |
| --- | --- | --- |
|  | 1.       Which author(s) conducted the interview? | Interviews conducted by PM |
|  | 2.       What were the researcher’s credentials? | MT: PhD in public health; CA: MD-PhD; SG: MD; AB: MD-PhD; ER: PhD |
|  | 3.       What was their occupation at the time of the study? | PM: PhD in public health; CA: professor of epidemiology; SG: researcher in public health; AB: university lecturer, researcher in public health ; ER: Research Director, researcher in public health |
|  | 4.       Was the researcher male or female? | 3 females, 2 males. The interviewer was a male |
|  | 5.       What experience or training did the researcher have | Experience in conducting qualitative research (PM, SG, ER), experience in interventional research surveys (PM, CA, AB), expertise in public health (all authors), expertise in sexual health (PM, AB, ER) |
|  | **Relationship with participants** | |
|  | 6.       Was a relationship established prior to study commencement | The interviewer did not know the participants before the study. |
|  | 7.       What did the participants know about the researcher? | The researcher introduced himself as a Phd fellow in Public Health.  At the start of the study, the aim of the research project, as well as the objectives of the study was presented. |
|  | 8.       What characteristics were reported about the interviewer/facilitator? | Only his level of education (Phd fellow) |
| **Domain 2: Study design** | **Theoretical framework** | |
|  | 9.       What methodological orientation was stated to underpin the study? | We used thematic analysis in a sociological theoretical approach. |
|  | **Participant selection** | |
|  | 10.   How were the participants selected? | All participants were recruited if they were between 15 and 24 years old, without distinction. We tried to represent a diversity of profiles and backgrounds. |
|  | 11.   How were the participants approached? | Originally from health promotion and education professionals. After by word of mouth, email, social networks sites. |
|  | 12.   How many participants were in the study? | 19 |
|  | 13.   How many participants refused to participate or dropped out? Why? | Not applicable: only volunteers contacted the interviewer. |
|  | **Setting** | |
|  | 14.   Where was the data collected? | On the phone (14 interviews) or in coffee shop (4 interviews) or in the interviewer’s office (1 interviews). |
|  | 15.   Was anyone else present besides the participants and researcher? | No. |
|  | 16.   What are the important characteristics of the sample? | Diversity of backgrounds, live places, identities (sexual and gender), and ages (see characteristics in tables 1 and 2) |
|  | **Data collection** | |
|  | 17.   Were questions, prompts, guides provided by the author? Was it pilot tested? | The interview guide was tested, read and adapted during the interview according to the expertise of each participant. |
|  | 18.   Were repeat interviews carried out? Details | No repeat interviews. |
|  | 19.   Did the researcher use audio or visual recording to collect the data? | All interviews recorded (only audio). |
|  | 20.   Were field notes made during and/or after the interview or focus group? | Notes taken during all interviews. |
|  | 21.   What was the duration of interviews or focus groups? | From 17-90 minutes. Average: 53 minutes |
|  | 22.   Was data saturation discussed? | Data saturation was discussed after 15 interviews. |
|  | 23.   Were transcripts returned to participants for comments and/or correction? | Transcripts not returned to participants |
| **Domain 3: Analysis and findings** | **Data analysis** | |
|  | 24.   How many data coders coded the data? | Two authors (PM, ER) created the initial coding tree using first samples interview. |
|  | 25.   Did authors provide a description of the coding tree? | The coding tree is the one presented in Table 3 and corresponds to the themes and sub-themes identified. Categories of proposals for health action are also available in Table 4. |
|  | 26.   Were themes identified in advance or derived from the data? | The themes were derived both inductively and deductively |
|  | 27.   What software, if applicable, was used to manage the data? | Use of NVivo software. |
|  | 28.   Did participants provide feedback on the findings? | No feedback was obtained from participants. |
|  | **Reporting** | |
|  | 29.   Were participant quotations presented to illustrate the themes/findings? Was each quotation identified? | We present some quotations to illustrate findings with relevant quotation identification (participant ID as referenced in Table 1). |
|  | 30.   Was there consistency between the data presented and the findings | The data presented and the findings are consistent. |
|  | 31.   Were major themes clearly presented in the findings? | We present the most important themes related to the study objectives in the findings. |
|  | 32.   Is there a description of diverse cases or discussion of minor themes? | We report and describe diverse cases. |
