# Supplementary material for: Young people’s proposals for a web-based intervention for sexual health promotion: a French qualitative study
Source: BMC Public Health. 2023 Jul 19;23:1389. doi: 10.1186/s12889-023-16257-8 (PMC10357632; doi:10.1186/s12889-023-16257-8)
Supplement: Supplementary file 2 — Supplementary material 2: Interview guide [file 12889_2023_16257_MOESM2_ESM.docx]

**Supplementary material 2**

**QUALITATIVE STUDY OF YOUNG PEOPLE'S PROPOSALS FOR PROMOTING SEXUAL HEALTH VIA THE INTERNET AND DIGITAL**

**INTERVIEW GUIDE**

**PRESENTATION**

Good morning/afternoon, my name is Philippe MARTIN, I'm a researcher at the Institut National de la Santé and I'm interested in sexuality education for young people, particularly as it relates to the Internet and social networks.

**BACKGROUND**

Before I begin, I'd like to remind you that our study focuses on how you seek information, your expectations in terms of sexuality education, and what you discuss on the Internet about sexuality, puberty, friendships and relationships, sexual orientation and sexually transmitted diseases. It can also be about screening or contraception. We want you to know that all the information you give us is strictly confidential and anonymous.

Before we start the interview, I would like to remind you that the interview will be recorded and the data collected will be totally anonymous and treated as confidential. You are free not to answer any of the questions, and you may withdraw from the study at any time. I would also like to point out that in the case of a vulnerable person in danger (physical, social, mental, etc.), whether a minor or an adult, it is compulsory to report the situation to the appropriate judicial or administrative authorities.

Do you agree to participate?

To take part, you need to confirm that you are not opposed to taking part in this interview by signing the information notice attesting to your non-objection to taking part in the study.

**PARTICIPANT CHARACTERISTICS**

- How old are you?
- What is your current educational or professional activity (course or job)?

**DIGITAL AND SEXUAL HEALTH CONTEXT**

**Internet and digital**

- Generally, what do you do on the internet, digitally?
- Which social networks, forums and blogs do you use, and why?
- Do you ever chat on social networks, forums or blogs, and with whom? With whom?
- What are the risks of using social media, and how do you protect yourself?

**Sexual health**

- In general, what questions do you have about sexuality/puberty/relationships/STDs/other (depending on age) ?
- Describe the exchanges you have with your friends about your questions about sexuality/puberty/relationships/STDs/other (depending on age)?
- Tell me about the last time you went online to answer these questions?
- What experiences do you share on the internet and social networks (about sexuality/puberty/relationships/STDs/other (depending on age))?

**CONCRETE PROPOSALS FOR ACTION**

- How do you feel about using the Internet and digital technology for sex education ?
- Do you think it's interesting for young people?
- Which digital and online media would you prefer?
- What features would you like to see developed?
- What sexual health topics/themes would you like to see addressed? How?
- What do you think of a platform on a website or on social networks to exchange with other young people or professionals on questions relating to sexuality or, more generally, health?
- If an online sexual health education initiative were developed, would you be interested in taking part?
